# Assessment of phytochemicals, antimicrobial and cytotoxic activities of extract and fractions from *Fagonia olivieri* (Zygophyllaceae)

**DOI:** 10.1186/1472-6882-13-167

**Published:** 2013-07-10

**Authors:** Umbreen Rashid, Muhammad Rashid Khan, Shumaila Jan, Jasia Bokhari, Naseer Ali Shah

**Affiliations:** 1 Department of Biochemistry, Faculty of Biological Sciences, Quaid-i-Azam University Islamabad, Islamabad 45320, Pakistan

**Keywords:** Antibacterial activity, Brine shrimps lethality assay, Cytotoxicity, *Fagonia olivieri*, Phytochemicals

## Abstract

**Background:**

In Pakistan *Fagonia olivieri* (Zygophyllaceae) is commonly used in the indigenous system of medicine for treatment of conditions like diabetes, cancer, fever, asthma, toothache, stomach troubles and kidney disorders. This study evaluated the crude methanol extract of *F. olivieri* (FOM) and its derived fractions for their antimicrobial and cytotoxic activities as well as the classes of phytochemical.

**Methods:**

Dried powder of whole plant of *F. olivieri* was extracted with methanol (FOM) and the resultant was fractionated to give *n*-hexane fraction (FOH), chloroform fraction (FOC), ethyl acetate fraction (FOE), *n*-butanol fraction (FOB) and residual aqueous fraction (FOA). Methanol extract and its derived fractions were subjected to phytochemical screening using standard procedures. Also the extract and fractions were assayed for antibacterial, antifungal and cytotoxic activities using agar well diffusion technique, agar tube dilution method and brine shrimps lethality test, respectively.

**Results:**

The results obtained for phytochemical analysis indicate the presence of saponins and alkaloids in all the tested extract and fractions while anthraquinones were not detected. The results showed that all the bacterial strains tested in this study were susceptible to at least one of the fractions tested. However, FOE and FOB were the best antibacterial fractions and showed antibacterial activity against maximum number of bacterial strains. The results showed that *Escherichia coli* was the most sensitive bacterium while *Bordetella bronchiseptica* and *Enterobacter aerogenes* were less susceptible against various fractions. Maximum percent inhibition for growth was recorded for the fungus *Aspergillus flavus* with FOE whereas growth of *Aspergillus fumigatus* and *Fusarium solani* was inhibited by FOM and its all derived fractions. Minimum LC_50_ (24.07 mg/L) for brine shrimp assay was recorded for FOE followed by LC_50_ of FOC (26.1 mg/L) and FOB (30.05 mg/L) whereas maximum LC_50_ was exhibited by FOH (1533 mg/L).

**Conclusion:**

These results indicated the use of *F. olivieri* to treat infections with emphasis to isolate and characterize the active principle responsible for antibacterial, antifungal and cytotoxic activities and its exploitation as therapeutic agent.

## Background

In many countries plants are used as a source of medicines to treat infections and other disorders and some of the most powerful and potent drugs used nowadays were derived from plants [1]. Various parts of plant (root, fruit, stem, flower, modified plant organs and twigs exudates) having different therapeutic properties are used as herbal medicines. They are collected on a minute scale to utilize by folk healers and local communities, while several others are collected as raw material in larger amounts to trade them in the market for numerous herbal industries [2].

In recent years a large number of plants have been investigated for their antimicrobial properties as an integrative system of medicine for protection and management against pathogens. Use of plant extracts having antimicrobial potential can be of great significance to treat human pathogenic diseases. Natural bioactive compounds in plants play a key role in plants defense system and are also well known for their unambiguous physiological action on human body. Amino acids, proteins common sugars and chlorophyll are plants primary metabolites whereas secondary metabolites comprise of flavonoids, alkaloids, tannins, saponins, and terpenoids. In view of the tremendous importance of secondary metabolites as therapeutic agents; they are becoming parts of the integrative health care system as alternative or supportive medicines [3].

The indiscriminate use of commercial antimicrobial drugs for curing infectious diseases has lead to the development of multiple drug resistance in human pathogenic microorganisms. In addition, a variety of side effects like allergic reactions, hypersensitivity and immune suppression are occasionally associated with antibiotics. Further, food preservation requires evaluation of natural resources such as herbal fractions and isolates with antimicrobial properties as the long historic use of herbs has proved their safety and efficacy in various traditional medicine systems. Recent trends for the use of natural remedies as antimicrobial have increased their use in food, cosmetic and pharmaceutical products which have been screened *in vitro* and indicated antimicrobial and other diverse properties.

Therefore there is a great interest for making new drugs and to avoid food spoilage by the development of novel effective compounds [4]. They would have vast therapeutic potential as they will mitigate many of the adverse effects that are often coupled with synthetic antimicrobials. One of the valuable means used to screen bioactive compounds from plant extracts is brine shrimp lethality assay [5]. It is considered as a convenient probe for primary evaluation of toxicity [6].

*Fagonia olivieri* belongs to the Zygophyllaceae family, which has about 25 genera and 240 species [7]. In Pakistan Zygophyllaceae family is represented by 8 genera and 22 species, whereby it is commonly found in dry hills areas of Khyber Pakhtoon Khawa and Baluchistan province. The chief genera of the family are *Fagonia, Zygophyllum, Gauiacum* and *Tribulus*[8]. Various members of this family are known for their medicinal uses. In our search for ethnomedicine, we found that extract of *F. olivieri* have been widely used for the management of diabetes and cough [9]. In addition it is described as a cooling agent and blood purifier [9]. In the indigenous system of medicine, aqueous decoction of aerial parts of *F. olivieri* is a popular remedy for the cancer in its early stages and for the treatment of various blood vascular and digestive system disorders [10]. The proposed pharmacological activities have not been reported for the whole plant extract of *F. olivieri*. The main objectives of the present study were to screen and evaluate antibacterial, antifungal, cytotoxic activities by using brine shrimps lethality assay and phytochemical analysis of crude methanol extract and its derived fractions.

## Methods

Whole plant of *F. olivieri* was collected from Rawalpindi Pakistan. Methanol, *n*-hexane, ethyl acetate, chloroform, *n*-butanol, sulphuric acid, hydrochloric acid, glacial acetic acid, ferric chloride, sodium hydroxide, benzene, ammonium hydroxide were purchased from Wako Co. (Osaka, Japan). Dimethyl sulphoxide (DMSO), roxythromycin, cefixime and terbinafine were procured from Sigma Co. (St. Louis, MO, USA). Nutrient agar and sabouraud dextrose agar was procured from Merck (Germany). Standard mild resistant isolates of bacteria; *Escherichia coli* ATCC 15224*, Pseudomonas aeruginosa* ATCC 27853, *Staphylococcus aureus* ATCC 6538, *Klebsiella pneumonia* MTCC 618, *Bordetella bronchiseptica* ATCC 4617, *Micrococcus luteus* ATCC 10240, *Enterobactor aerogenes* ATCC 13048 and fungi; *Aspergillus niger, Aspergillus flavus, Aspergillus fumigatus, Fusarium solani,* and *Mucor piriformis* were obtained from Laboratory of Molecular Biology Department of Biochemistry Quaid-i-Azam University Islamabad, Pakistan.

### Collection, extraction and fractionation of plant materials

Whole plant samples of *F. olivieri* were collected from Dhamyal Rawalpindi, Pakistan in May, 2010 and identified by Dr. Saleem Ahmad, Principal Scientific Officer, Pakistan Museum of Natural History, Islamabad. Voucher specimen was deposited at Pakistan Museum of Natural History (Voucher No. 058608).

Plant samples were collected in cotton bags and dried under shade for one month. The material was ground using a grinder into a fine powder. Two kilogram of ground material was soaked in 4 L of methanol for one week and filtered. The material was again extracted with 4 L of methanol and filtered after one week. Two percolates were mixed and dried under vacuum in a rotary evaporator to obtain methanol extract of *F. olivieri* (FOM). Four gram of the crude extract was suspended in 200 ml of distilled water and partitioned with *n*-hexane (FOH), chloroform (FOC), ethyl acetate (FOE), *n*-butanol (FOB) and residual aqueous fraction (FOA).

### Determination of the phytochemical constituents

The FOM and its derived fractions were evaluated for the occurrence of flavonoids, tannins, saponins, phlobatannins, cardiac glycosides, alkaloids, terpenoids and anthraquinone using simple qualitative methods.

#### Salkowski test for terpenoids

To a 5 ml of extract and each fraction 2 ml of chloroform was added and followed by addition of 3 ml of concentrated H_2_SO_4_. At interface a reddish brown coloration is formed if terpenoid constituent is present [11].

#### Test for alkaloids

Exactly 0.5 g of extract and each fraction was warmed with 8 ml of 1% HCl and filtered. Two milliliters of each filtrate was titrated separately with (a) Mayer’s reagent and (b) Dragendroff’s reagent. The presence of alkaloids was indicated by the formation of cream or yellow precipitate [11].

#### Test for saponins

About 0.2 g of the crude extract and each fraction was added to 20 ml of distilled water and boiled in a water bath and filtered. An aliquot of 5 ml of distilled water was mixed with 10 ml of the filtrate and shake it vigorously. Formation of emulsion was observed after mixing the frothing with 3 drops of olive oil and shaken vigorously [11].

#### Test for phlobatannins

In a small tube 80 mg of crude extract and each fraction was boiled in 1% aqueous hydrochloric acid; formation of red precipitate indicated the presence of phlobatannins [12].

#### Keller- Kiliani test (for de-oxy sugars in cardiac glycosides)

From the crude extract and each fraction, 5 ml of aqueous extract (10 mg/ml) was mixed with 2 ml of glacial acetic acid having a drop of ferric chloride solution followed by 1 ml of concentrated H_2_SO_4_. The characteristic feature of deoxysugars in cardenolides is the formation of brown ring at interphase [12].

#### Test for coumarins

About 0.3 g of extract and each fraction was taken in a test tube. Filter paper moistened with 1 N NaOH solution was used to cover the mouth of the tube. Test tube was placed in boiling water for few minutes. Filter paper was removed to examine under UV light. The presence of coumarins is indicated by yellow fluorescence [12].

#### Test for anthraquinones

Briefly, 1.0 g of extract and each fraction was boiled in 1% HCl and filtered. To the filtrate 5 ml of benzene was added and in the benzene layer after shaking 10% NH_4_OH was added and color in the alkaline phase was observed. Formation of pink/violet or red color indicated the presence of anthraquinones [12].

#### Test for tannins

About 0.5 g of the extract and each fraction was mixed with 20 ml of water in a test tube and filtered. Formation of blue black or brownish green color after treating filtrate with a few drops of 0.1% ferric chloride indicated the presence of tannins [13].

#### Test for flavonoids

A portion of the aqueous filtrate of extract and each fraction was mixed with 5 ml of dilute ammonia solution and concentrated H_2_SO_4_ was added. A yellow coloration is observed if flavonoid compounds are present [13].

### Antimicrobial assays

#### Preparation of bacterial/fungal inoculums

Four isolated colonies were inoculated in the 30 ml nutrient broth and incubated for 24 h at 37°C so that the growth in the broth was equivalent with McFarland standard (0.5%).

#### Antibacterial activity

Antibacterial activity of plant extract and its derived fractions was investigated by agar well diffusion method [14] using nutrient agar medium. Exactly 2 g of nutrient agar was dissolved in 100 ml of distilled water (pH 7.0) and was autoclaved. It was cooled down to 45°C. Then 100 ml of this media was seeded with 1 ml of inoculum having size of 10^6^ CFU/ml as per McFarland standard and after proper homogenization 75 ml was poured into the petri plate of 14 cm diameter. For agar well diffusion method, eleven wells per plate were made with the help of a sterile cup-borer (8 mm). Extract and fractions at concentration of 25, 15, 12.5, 10, 7.5, 5, 3, 2 and 1 mg/ml were prepared in DMSO. The test samples (100 μl) were poured into the wells and petri plates were then incubated at 37°C for 24 h. Simultaneously, roxythromycin and cefixime were used as positive controls whereas DMSO was used as a negative control. The lowest concentration inhibiting growth was taken as the minimum inhibitory concentration (MIC).

#### Antifungal activity

Agar tube dilution method was used to evaluate the antifungal activity [15]. The stock solution of extract, fractions and positive control; terbinafine were prepared by dissolving the extract and fractions in DMSO at a concentration of 12 mg/ml. The negative control was DMSO. Media for fungus was made by dissolving 6.5 g of sabouraud dextrose agar in 100 ml of distilled water (pH 5.6). Then 4 ml of it was added into screw cap tubes. These tubes were autoclaved for 15 min at 120°C and then cooled down to 45°C. The stock solution (66.6 μl) was mixed with media to give the final concentration of 200 μg/ml of sabouraud dextrose agar. Tubes were then solidified in the slanted position at 25°C. An agar surface streak was employed by inoculating each tube with a piece (4 mm diameter) of inoculum taken from a seven days old culture of fungi. Fungal growth inhibition was observed visually after 7 days of incubation at 28 ± 1°C. The percent inhibition of growth was calculated with reference to the negative control.

### Brine shrimp lethality assay

#### Hatching of brine shrimps

Brine-shrimp eggs *(Artemia salina)* were used to find out the cytotoxicity of the extract and various fractions. Hatching tray was half filled with saline solution (28 g sea commercial salt, Sigma USA, was dissolved in one litre of distilled H_2_O) and shrimp eggs were sprinkled into it. The tray was placed at room temperature (25 – 29°C) in the presence of florescent lamp for 24 h. Eggs were hatched after incubation and the test samples were used for cytotoxicity potential of the extract and fractions.

#### Assay procedure

This assay was performed by using the method of Meyer et al. [16]. Different concentrations of the extract and fractions; 1000 mg/L, 100 mg/L and 10 mg/L were prepared in DMSO and used against brine shrimp larvae. The survival rate of these larvae was observed against all concentrations of the extract and different fractions. For this purpose, 0.5 ml sample of each fraction was transferred in 20 ml vial, followed by the addition of 2 ml of sea commercial salt water. Ten shrimps transferred into each vial, final volume was adjusted to 5 ml by artificial seawater. All the vials were incubated for 24 h under florescence light at room temperature (25–29°C) and the survivors were counted with the help of magnifying glass. Percentage mortality was determined for three replications. LC_50_ values were calculated by using probit analysis of Biostat software (version 2009).

## Results and discussion

Ethnopharmacology is the most common approach used for selecting the plants for pharmacological studies [17]. Since different plant parts can be used to cure diarrhoea, cough, cold, fever bronchitis, cholera etc.; therefore they are proved to be a significant source of potentially helpful structures for the development of novel chemotherapeutic drugs [18,19]. In the present era, due to the onward march of civilization the plentiful resources of medicinal plants are declining rapidly [20]. The health related matters of rural population of Pakistan still mainly depends on the indigenous system of medicine [21]. Keeping in view the traditional uses of the plant, we carried out *in vitro* biological screening of methanol extract and its derived fractions of herbal medicine *F. olivieri.* It is well known that the bioactive phytocomponents are responsible for the medicinal value of the plants [22]. Hence the study of plant constituents can help explain scientifically the traditional use of these plants.

The results of the phytochemical analysis indicated the existence of saponins, alkaloids, cardiac glycosides, flavonoids and tannins in FOM and all other fractions. However cardiac glycosides were not determined in FOC and FOB. The plant constituents of FOM herein presented was instigated by other reports where saponins, alkaloids, cardiac glycosides, flavonoids and tannins have been detected in methanol extract of *Fagonia cretica*[23]. Presence of anthraquinones was not established in FOM and in all other fractions. Results obtained in this study are in line to other reports where anthraquinones were not detected in *F. cretica*[23]. Phlobatannins were only present in FOM and FOE fraction. However, terpenoids were detected in FOM and in all fractions except in FOC fraction (Table 1). Similar plant constituents have been detected in *Fagonia indica*[24,25].

**Table 1:** **Phytochemical constituents of *F. olivieri *methanol extract and its fractions**

**Phytochemical constituents**	**Extract and fractions**					
	**FOM**	**FOH**	**FOC**	**FOE**	**FOB**	**FOA**
Tannins	+	-	-	-	-	+
Saponins	+	+	+	+	+	+
Flavonoids	+	-	-	-	+	+
Alkaloids	+	+	+	+	+	+
Cardiac glycosides	+	+	-	+	-	+
Terpenoids	+	+	-	+	+	+
Phlobatannins	-	-	-	+	-	-
Coumarins	+	-	-	+	+	-
Anthraquinones	-	-	-	-	-	-

+, present; -, absent.

*FOM*; *F. olivieri* methanol extract, *FOH*; *F. olivieri n*-hexane fraction, *FOC*; *F. olivieri* chloroform fraction, *FOE*; *F. olivieri* ethyl acetate fraction, *FOB*; *F. olivieri n*-butanol fraction, *FOA*; *F. olivieri* residual aqueous fraction.

The systemic screening of plant extracts for antibacterial activity is a continuous effort to find new antibacterial compounds. The test organisms used in this study are related with diverse forms of human infections. From a medical viewpoint, *E. coli* results into septicemia and infection of lungs, gall bladder, skin lesions and meninges, and also a number of food related diseases that manifest themselves in the form of diarrhea [26,27]. *S. aureus* causes boil, ulcers, food poisoning, toxic shock and pneumonia etc.

Methanol extract and its all derived fractions of *F*. *olivieri* were tested at various concentrations (1.0 ~ 25.00 mg/ml), against *E. coli* ATCC 15224*, P. aeruginosa* ATCC 27853, *S. aureus* ATCC 6538, *K. pneumonia* MTCC 618, *B. bronchiseptica* ATCC 4617, *M. luteus* ATCC 10240, *E. aerogenes* ATCC 13048 and the evaluated MIC values are reported in Table 2. Methanol extract of *F. olivieri* and FOH exhibited low level of minimum inhibitory concentration (MIC) against *E. coli* (MIC = 3 mg/ml) while moderate level of MIC was manifested against *M. luteus* (MIC = 7.5 mg/ml). However, MIC values for FOM and FOH were 12.5 mg/ml and 15.0 mg/ml for *K. pneumonia*, respectively. Similarly *S. aureus* and *P. aeruginosa* were reported the most susceptible bacteria against the methanol extract of *F. cretica*[23].

**Table 2:** **The MIC values of *F. olivieri *crude extract and fractions**

**Bacteria**	**Minimum inhibitory concentration (mg/ml)**							
	**FOM**	**FOH**	**FOC**	**FOE**	**FOB**	**FOA**	**Roxithromycin**	**Cefixime**
*Staphylococcus aureus*	10	5	-	3	-	-	0.2	0.02
*Micrococcus luteus*	7.5	7.5	7.5	3	15	15	0.06	0.05
*Escherichia coli*	3	3	3	3	5	2	0.14	0.03
*Klebsiella pneumoniae*	12.5	15	7.5	5	10	25	0.17	0.13
*Bordetella bronchiseptica*	-	-	-	-	3	-	0.14	0.16
*Enterobactor aerogenes*	-	-	-	-	5	-	0.16	0.15
*Pseudomonas aeruginosa*	3	15	15	12.5	10	12.5	0.14	0.12

- = not active against tested microorganism.

*FOM*; *F. olivieri* methanol extract, *FOH*; *F. olivieri n*-hexane fraction, *FOC*; *F. olivieri* chloroform fraction, *FOE*; *F. olivieri* ethyl acetate fraction, *FOB*; *F. olivieri n*-butanol fraction, *FOA*; *F. olivieri* residual aqueous fraction.

Similarly FOE showed low level of MIC against *S. aureus*, *E. coli*, *M. luteus* (MIC = 3 mg/ml) and *K. pneumoniae* (MIC = 5 mg/ml); whereas MIC value recorded against *P. aeruginosa* was 12.5 mg/ml and no activity was detected against rest of the strains. Likewise FOC, FOB and FOA had low level of MIC values against *E. coli*, while higher MIC values were recorded for *K. pneumoniae*.

Antibacterial activities of plant extracts can be explained on the basis of their chemical constituents. From the results it is depicted that *E. coli* is the most susceptible while the *K. pneumoniae* and *P. aeruginosa* were less susceptible. Similar results have been determined for various extracts of *F. indica*[25]. Presence of different bioactive constituents; saponins, flavonoids and alkaloids in the extract and various fractions might contribute towards diversified antibacterial activities.

Persistent opportunistic fungal infections have become an important factor for morbidity and mortality in immune compromised patients [28]. The majority of the fungal infections are mainly caused by the *Aspergillus* species [29]. Therefore the plant extract and all fractions have been screened for antifungal activity against *A. niger, A. flavus, A. fumigatus, F. solani,* and *M. piriformis* using agar tube dilution method. The drug used as a standard was terbinafine.

The growth inhibition for the extract and fractions was measured and represented in Table 3. FOB and FOA did not show any inhibition in growth of *A. niger* while maximum inhibition was exhibited by FOH followed by FOE, FOC and FOM. *Aspergillus flavus* was the most sensitive fungus in this study with highest growth inhibition (78.9%) was recorded for FOE fraction. However, *M. piriformis* was less susceptible fungus and its growth was inhibited (11.8%) by FOH and FOC fraction.

**Table 3:** **Antifungal activity of the crude extract and various fractions of *F. olivieri***

**Fungi**	**Percent inhibition (%) (200 μg/ml)**						**Positive control**
	**FOM**	**FOH**	**FOC**	**FOE**	**FOB**	**FOA**	**Terbinafine (200 μg/ml)**
*Aspergillus niger*	15	42.1	25	36.8	0	0	100
*Aspergillus fumigatus*	30.8	16.6	33.3	25	41.7	35	100
*Aspergillus flavus*	50	31.6	0	78.9	40	0	100
*Fusarium solani*	22.2	35	30	5.2	15	55.5	100
*Mucor piriformis*	0	11.8	11.8	0	0	0	100

*FOM*; *F. olivieri* methanol extract, *FOH*; *F. olivieri n*-hexane fraction, *FOC*; *F. olivieri* chloroform fraction, *FOE*; *F. olivieri* ethyl acetate fraction, *FOB*; *F. olivieri n*-butanol fraction, *FOA*; *F. olivieri* residual aqueous fraction.

Brine shrimps cytotoxicity assay has been considered as pre-screening assay for antimicrobial, antitumor, antimalarial and insecticidal activities. Therefore it is suggested to be a convenient probe for the pharmacological activities of plant extracts [30]. The brine shrimps lethality assay was used to assess the toxicity of *F. olivieri*. The test samples (crude and fractions) were used in concentrations of 10, 100 and 1000 mg/L.

Results for the lethality were noted in terms of deaths of larvae (% age mortality) of brine shrimp. The FOM showed cytotoxic activity (LC_50_ = 356.4 mg/L) for the experimental shrimps. Different fractions exhibited varied cytotoxic activities. The LC_50_ value calculated was 1533 mg/L for FOH whereas LC_50_ for FOE was 24.07 mg/L. The FOC showed LC_50_ = 26.1 mg/L for brine shrimp lethality assay. Similarly, the FOB exhibited LC_50_ value of 30.05 mg/L whereas 101.2 mg/L was determined for FOA. The cytotoxic results of the samples are presented in Table 4.

**Table 4:** **Illustration of % age mortality of brine shrimps at different concentrations of extract and fractions and respective LC**_**50 **_**values**

**Extracts**	**% Mortality at various concentrations**			
	**1000 mg/L**	**100 mg/L**	**10 mg/L**	**LC**_**50 **_**(mg/L)**
FOM	56.66	41.66	27.66	356.4
FOH	47.66	31.33	17.66	1533
FOC	78.66	67.66	38.66	26.10
FOE	98.66	74.33	47.66	24.07
FOB	85.33	64.33	37.66	30.05
FOA	72.33	48.66	28.66	101.2

*FOM*; *F. olivieri* methanol extract, *FOH*; *F. olivieri n*-hexane fraction, *FOC*; *F. olivieri* chloroform fraction, *FOE*; *F. olivieri* ethyl acetate fraction, *FOB*; *F. olivieri n*-butanol fraction, *FOA*; *F. olivieri* residual aqueous fraction.

The spectra of antimicrobial activities displayed by the extract and fractions could perhaps be explained by the presence of different plant constituents. The mode of action of antibacterial effects of saponins may involve damage to the membrane causing leakage of cellular materials, finally leading to cell death [31]. This antimicrobial activity of *F. olivieri* may be correlated well with the presence of saponins. Similar findings have been reported for antimicrobial and cytotoxicity studies in other reports [23,25].

Flavonoids and tannins are effective antimicrobial substances possibly due to their capability to interact with soluble and extracellular proteins and to intricate with bacterial cell walls leading to the death of the bacteria [32]. The results presented in this study were concurrent with the above findings. Similar antimicrobial results have been reported in earlier studies [23,25].

## Conclusion

The result of this study signifies the potential of *F. olivieri* as a source of therapeutic agent. Further studies on the purification of bioactive components can depict the exact potential(s) of the plant to restrain a number of pathogenic microbes as the purified components may have even more effectiveness with respect to inhibition of microbes. Furthermore, the activity exhibited by the extracts for brine shrimps and microbes that are associated with various infectious diseases, may offer scientific justification for the ethnomedicinal use of the plant.

## Competing interests

The authors declare that they have no competing interests.

## Authors’ contributions

UR made significant contribution to acquisition of data, analysis, drafting of the manuscript. MRK has made substantial contribution to conception and design, interpretation of data, drafting and revising the manuscript for intellectual content. SJ, JB and NAS participated in the design and collection of data and analysis. All authors read and approved the final manuscript.

## Pre-publication history

The pre-publication history for this paper can be accessed here:

http://www.biomedcentral.com/1472-6882/13/167/prepub
